# Evaluation of the Effect of Aqueous* Momordica charantia* Linn. Extract on Zebrafish Embryo Model through Acute Toxicity Assay Assessment

**DOI:** 10.1155/2019/9152757

**Published:** 2019-05-02

**Authors:** Siroshini K. Thiagarajan, Khamini Rama Krishnan, Thandar Ei, Nurul Husna Shafie, Daryl J. Arapoc, Hasnah Bahari

**Affiliations:** ^1^Department of Human Anatomy, Faculty of Medicine and Health Sciences, Universiti Putra Malaysia, 43400 Serdang, Selangor, Malaysia; ^2^Department of Nutrition and Dietetics, Faculty of Medicine and Health Sciences, Universiti Putra Malaysia, 43400 Serdang, Selangor, Malaysia; ^3^Malaysian Nuclear Agency, 43600 Kajang, Selangor, Malaysia

## Abstract

*Momordica charantia* Linn., commonly known as bitter gourd, has many protective roles due to its medicinal value as it contains bioactive components. However, this extract showed possible toxicity effect on zebrafish embryo. Thus this study was designed to differentiate the toxicity activities in two types of* M. charantia* sample which are Indian and Chinese* M. charantia*, as well as to compare between two different aqueous extraction methods, hot and cold aqueous method, using zebrafish embryo assay assessment. It was observed that the survival rate of zebrafish embryo decreased as the concentration of test extract increased for all samples of* M. charantia*. The LC_50_ values of hot aqueous Chinese* M. charantia*, hot aqueous Indian* M. charantia*, and cold aqueous Chinese* M. charantia* were 144.54 *μ*g/ml, 199.53 *μ*g/ml, and 251.19 *μ*g/ml, respectively. However, cold aqueous Indian* M. charantia* has a higher LC_50_ which was not in the range of the tested concentration. Hatchability of* Danio rerio* embryo reduced as the concentration of* M. charantia* extract increased while no hatching was observed in the highest concentration (1000 *μ*g/ml). Scoliosis of zebrafish larvae was only seen in higher concentrations (125-1000 *μ*g/ml) of extract. The heartbeat of zebrafish larvae treated with* M. charantia* extract was within the normal range, 120-180 bpm, but at higher concentrations (125-1000 *μ*g/ml) the heartbeat differed for all samples of test extract. Hence, although this plant extract was safe to be consumed due to its pharmaceutical effect, it still exhibited mild toxicity effect at higher concentration when it was evaluated on zebrafish embryo.

## 1. Introduction

Traditional medicine or folk medicine is a complementary and alternative medicine which is widely used in many countries. It gained popularity because people believe that traditional medicine has beneficial components which are made up of natural products. Based on their own mechanism of action, these natural sources can treat various diseases like allopathic drugs. Therefore, the main difference between conventional and folk medicine is that the former one is chemically treated and scientifically proven safe to be consumed. With conventional medicine the diagnosis is based on symptoms, whereas the folk medicine has a preventive approach as it is obtained from natural sources [1]. Despite minimal side effect due to a small amount of chemicals, heavy metals in the traditional medicine can eventually cause toxic effect over a high and prolonged consumption [2].

The toxic agent is mostly derived from sources like plants, animals, and microorganisms. As a toxic agent, it transmits the toxic substance through the various modes of transmission mainly via direct contact. A toxicology test is necessary, not only for allopathic medicine but also for complementary and alternative medicine to discover any adverse effects which are not known until the signs and symptoms develop upon high consumption. Some common adverse effects of traditional medicine include nausea, vomiting, weakness, dizziness, hypotension, paraesthesia of mouth and tongue, arrhythmia, and ventricular fibrillation [3]. Assessment of toxicity using toxicity testing assays can prove the safety of traditional medicine and promote its consumption.

Toxicity testing is accomplished to screen the chemical compositions of a sample to ensure that it contains no life-threatening components [4]. Furthermore, the importance of toxicity testing is to provide a dose-responsive curve against the toxicity effect, study the safety of components in the sample, and authenticate methods of investigating toxicity [5]. Zebrafish (*Danio rerio)* and its transparent embryos are among the apt models used to study the toxicity effect on the embryonic development. It is used to inspect the bioactive compounds of the sample through toxicity assay. Commonly used mammalian models have some drawbacks with higher cost and prolonged time for results, being yet ethically questionable. Moreover, genetically humans show great similarities of genomic sequences and brain patterning with the* Danio rerio*. Thus, this makes zebrafish embryo an advantageous assay in exploring many diversions of toxicology study yielding a prompt outcome [4].

*Momordica charantia* Linn., locally known as bitter gourd or bitter melon, is a type of vegetable-fruit used vastly as ethnomedicine. It is found in tropical regions, for instance, China, India, Tropical Africa, America, and Thailand. Bitter gourd has many medicinal values including antidiabetic, hypoglycemic, antitumor, antioxidant, antiulcer, analgesic, and antifertility properties [6]. The bioactive component in this unique fruit is answerable for the therapeutic properties.

There are two different types of bitter gourds, which are the Chinese and Indian bitter gourd with their own specialty. Bitter gourd is mostly eaten raw or with preservation in water to ensure that the medicinal components of the bitter gourd is preserved. It can be consumed by using hot or cold water depending on the individual's preference. For example, it is stated that, in China, the community consumed bitter gourd in combination with tea or hot water upon fermentation as it is believed that the bitter gourd has more natural weight losing properties than other allopathic drugs [7]. The cold bitter gourd is consumed as bitter gourd juice in combination with other fruits as well as vegetables.

Even with the increase in bitter gourd products which have been marketed, there are many cases of the adverse effect of bitter gourd consumption as it has the tendency of producing toxic effect. Thus, the aim of this study was to evaluate the effects of aqueous* Momordica charantia* Linn. exposure on zebrafish embryo through acute toxicity assay assessment.

### 1.1. Zebrafish Embryo Model

Zebrafish, scientifically known as* Danio rerio*, is a vertebrate animal model which is used widely in the field of Biomedical Science for toxicology testing [8]. It is used as an alternative animal model due to its advantages. Generally, the wide usage of zebrafish embryo is attributed to its short life cycle that produces rapid and prominent results [8]. The zebrafish embryo has fast embryogenesis process and produces a large number of eggs. Moreover, these eggs are highly permeable and highly tolerant to the penetration of chemical components. The embryo is transparent enough to observe the development of embryogenesis easily using the standard dissecting light microscope [9]. Some of the main parameters which are focused on during the toxicity assessment are survival rate, LC_50_ determination, hatching rate, scoliosis rate, and heart rate of the zebrafish embryo.

The survival rate refers to the ability of an organism to adapt in a particular environment according to its optimal condition requirement. Some of the ideal conditions which can affect the survival rate of zebrafish embryo include pH, temperature, chemistry, and hardness of water, as well as nitrogenous waste factor [10]. Higher survival shows that the zebrafish embryo is able to withstand the specific environment whereas low survival rate indicates that the embryo is unable to endure the surrounding atmosphere. The median lethal concentration was obtained from the mortality rate of the zebrafish embryo, in order to identify the safe concentration level for consumption of each* M. charantia* sample. In general, the higher LC_50_ values indicate that the toxicity level is low because higher concentration is needed to result in 50% of mortality rate of an organism, whereas a lower LC_50_ points out that the toxicity of the sample is more harmful because in lower concentration there is greater death of organism [10].

The zebrafish embryo has an external fertilization whereby the eggs will be deposited from the zebrafish yolk sack during the spawning process. The nonadherent embryo will then normally hatch within 48-72 hours post-fertilization [11]. The embryo is sensitive and has specific consideration for its development. The unfavorable condition can literally cause stress to the embryo affecting its growth and development stage resulting in morphological abnormalities [12]. One of the common morphological abnormalities is scoliosis. Scoliosis is the abnormal curvature of the spine causing the spine to have a “C” or “S” like shape. The scoliosis is basically a type of teratogenic effect. It is influenced by a few surrounding stress factors such as chemical compounds and heavy metals [13]. The presence of scoliosis literally indicates that the sample is toxic. The heartbeat of a developing wild-type* Danio rerio* begins at 36hpf [14]. The normal range of zebrafish heartbeat is between 120 and 180 beats per minute. Additionally, the heartbeat of zebrafish can be influenced by chemical factors, genetic-induced alteration, temperature, and many others [15]. These factors cause the heartbeat rate to alter resulting in bradycardia or tachycardia depending on the particular factor manipulating the zebrafish model.

## 2. Methods

### 2.1. Sample Collection

The Chinese* M. charantia *(C) and Indian* M. charantia* (I) samples were collected in Selangor Malaysia. Both plants were identified and authenticated by Institute of Bioscience, UPM (Plant Voucher Number: Chinese Bitter Melon: SK3160/17; Indian Bitter Melon: SK3157/17). The fresh fruit of* M. charantia* was sliced and oven-dried at 40 degrees Celsius (40°C) [16]. This was followed by powdering the dried sample.

### 2.2. Sample Preparation

*Momordica charantia *L. extraction was carried out using hot and cold aqueous extraction method for both types of* M. charantia*. The ground sample of* M. charantia *weighing 50 g was placed into a conical flask containing about 500 ml of distilled water. The dilution was based on the standard solvent to sample ratio of extraction preparation which is 1:10 [17]. Next, the diluted* M. charantia *sample in the conical flask was wrapped with an aluminum foil and placed in the water bath at 70°C for 24 hours [18]. Hot samples were abbreviated as hot aqueous (HA). As for the cold aqueous method, it was accomplished by keeping it at room temperature for 2 days [19]. Cold samples were abbreviated as cold aqueous (CA). The sample was then filtered using Whatman paper 1 and was placed at -20°C for freeze-drying in order to preserve and extend the shelf life of the sample collected.

### 2.3. Serial Dilution

Twofold serial dilution method was used to obtain a series of* M. charantia* extract samples with different concentrations. The stock solution was prepared by diluting 0.1 gram of* M. charantia* extract with 1000 *μ*l of distilled water. From the stock solution, six successive series of concentration were produced, which were 15.625 *μ*g/ml, 31.25 *μ*g/ml, 62.5 *μ*g/ml, 125 *μ*g/ml, 250 *μ*g/ml, and 500 *μ*g/ml. On the other hand, as for the control treatment, 500 mg dose of paracetamol was used as a positive control, and distilled water was used as a negative control. The paracetamol sample was prepared through serial dilution using a similar method, producing the same series of concentration as the test extract [20].

### 2.4. Zebrafish Embryo Assay

One zebrafish embryo was transferred into each of the 96-well plates using a pasteur pipette at 24 hours post-fertilization (24hpf) [21]. Next, the treatment was given by pipetting the sample into each well in the 96-well plates based on different series of concentrations. The embryo development was observed after 24, 48, and 72 hours of treatment. The zebrafish embryo was viewed under the inverted microscope by focusing on a few parameters such as the survival rate, hatching rate, scoliosis, and heartbeat [22]. In addition, the number of heartbeats was counted for 15 seconds and then it was quantified by multiplying by four, to get the number of heartbeats per minute [23].

### 2.5. Data Analysis

The linear regression and one-way Analysis of Variance (ANOVA) were used to analyze the data obtained. The lethal concentration at 50% (LC_50_) of each* M. charantia* extract was calculated, through the linear regression graph of mortality rate in probit unit against the log concentration [24]. The one-way ANOVA was used to analyze the types of* M. charantia* extract affecting the survival rate, hatching rate, scoliosis, and heartbeat of zebrafish embryo influenced by different series of concentrations. Finally, all the data were interpreted as mean±SEM with a* p* value of <0.05 indicating the result to be statistically significant based on the statistical hypothesis testing used.

## 3. Results

### 3.1. Effect of* M. charantia* Extract on Survival Rate of Zebrafish Embryo

**Figure 1:**
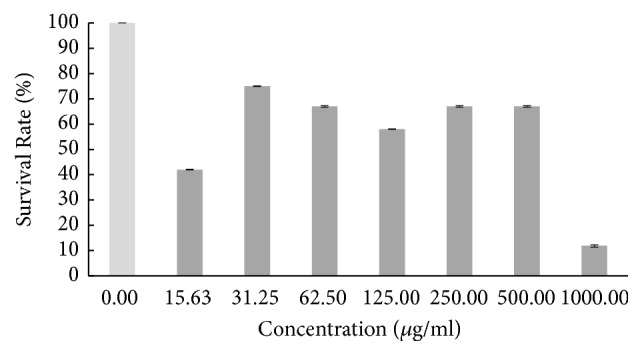
The survival rate of zebrafish embryo examined on hot aqueous Chinese* M. charantia *(CHA) at different series of concentration. Results were expressed as mean ± SEM of three independent experiments performed in triplicate.

**Figure 2:**
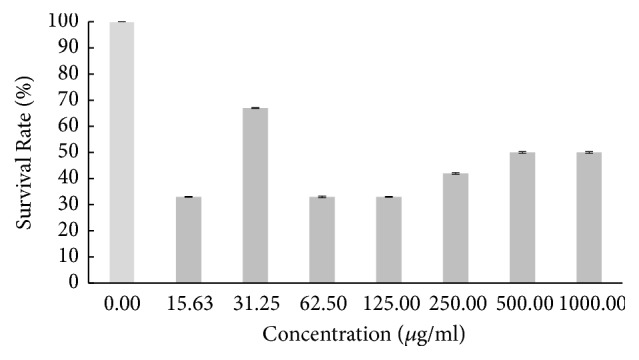
The survival rate of zebrafish embryo examined on hot aqueous Indian* M. charantia* (IHA) at different series of concentration. Results were expressed as mean ± SEM of three independent experiments performed in triplicate.

**Figure 3:**
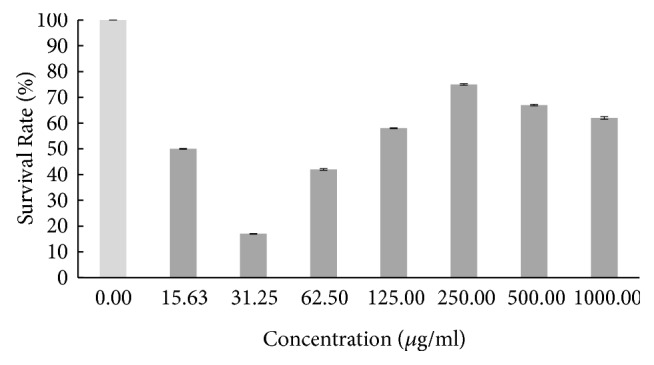
The survival rate of zebrafish embryo assessed on cold aqueous Chinese* M. charantia* (CCA) at various series of concentration. Results were expressed as mean ± SEM of three independent experiments performed in triplicate.

**Figure 4:**
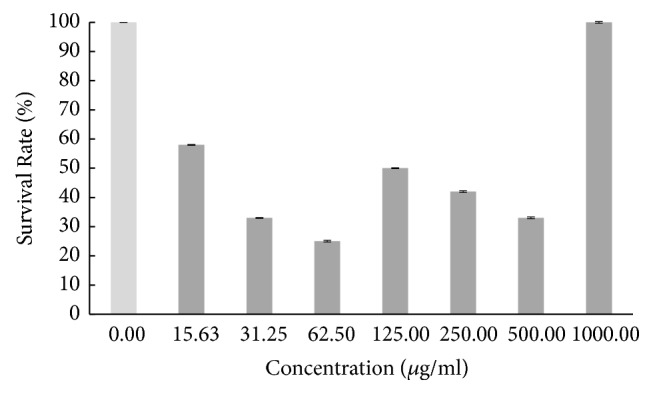
The survival rate of zebrafish embryo examined on cold aqueous Indian* M. charantia* (ICA) at different series of concentration.

**Figure 5:**
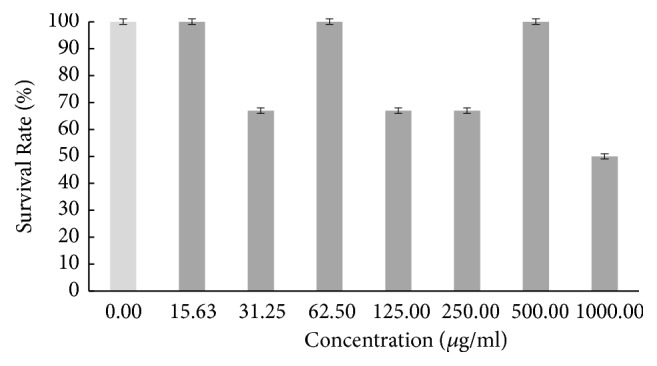
The survival rate of zebrafish embryo tested on paracetamol at different series of concentration. Results were expressed as mean ± SEM of three independent experiments performed in triplicate.

Based on the result in Figure 1, the survival rate of the zebrafish embryo was fluctuating as the concentration of hot aqueous Chinese* M. charantia* extract increased, yet it still exhibited a lower overall survival rate. The survival rate of embryo tested on hot aqueous Indian* M. charantia* extract showed that there was an inconsistent reduction as the concentration increased (Figure 2), followed by the survival rate of zebrafish embryo which gradually increased initially (31.25 *μ*g/ml), and after that there was slight constant decline observed at higher concentration (250-1000 *μ*g/ml) for cold aqueous Chinese* M. charantia* (Figure 3). It is also shown that the survival rate of embryo inconsistently decreased as the concentration of cold aqueous Indian* M. charantia* extract increased but a 100% survival rate was observed in the highest concentration (1000 *μ*g/ml) (Figure 4). As for the paracetamol, the survival rate decreased as the concentration increased, but the overall survival rate was higher compared to all the test extracts (Figure 5).

### 3.2. Effect of* M. charantia* Extract on Lethal Concentration at 50% of Survival (LC_50_) Values Assessed on* Danio rerio* Embryo Model

**Figure 6:**
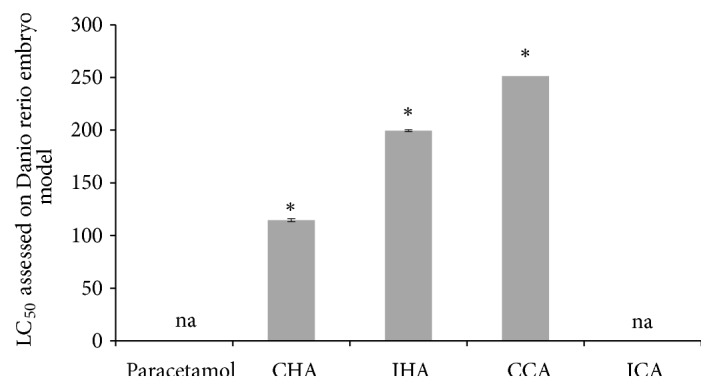
The lethal concentration at 50% of survival (LC_50_) values assessed on* Danio rerio* embryo model. Results were expressed as mean ± SEM of three independent experiments performed in triplicate. ^#^*p*<0.05 compared with paracetamol.

The LC_50_ value of hot aqueous Chinese* M. charantia* was 144.54 *μ*g/ml and the extract with lower concentration than this value was considered safe to be consumed for the zebrafish embryo. For the hot aqueous Indian* M. charantia*, the concentration was below 199.53 *μ*g/ml which was considered safe to be consumed by the zebrafish embryo. Furthermore, the LC_50_ of cold aqueous Chinese* M. charantia *was 251.19 *μ*g/ml, more than that of the cold aqueous Indian* M. charantia* that was unavailable as the LC_50_ was out of the tested range. Paracetamol also showed unavailable data which is higher than the tested concentration, which indicates that it exhibits a lower toxicity effect (Figure 6).

### 3.3. Effect of* M. charantia* Extract on Hatching Rate of* Danio rerio* Embryo

**Figure 7:**
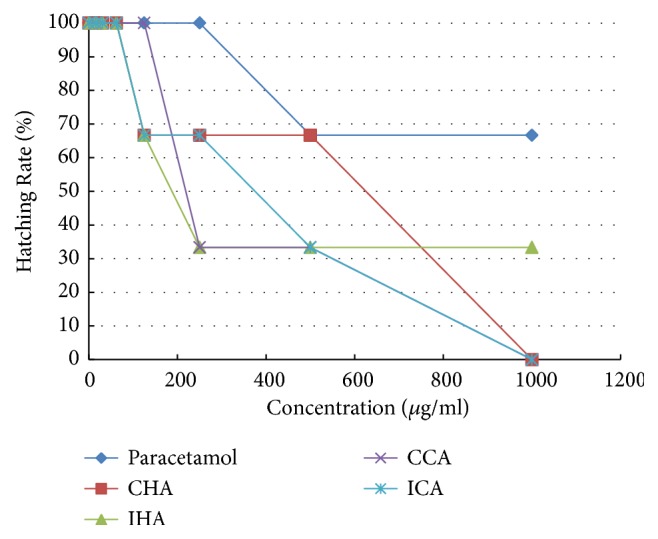
The hatching rate of zebrafish embryo examined on all test extracts and control solutions at various series of concentration. Results were expressed as mean ± SEM of three independent experiments performed in triplicate.

According to the result obtained (Figure 7), the zebrafish embryo showed a delayed hatchability in all the samples of* M. charantia* test extract as the concentration of test extract increased. At lower concentration (0-62.5 *μ*g/ml) of treatment solution, the hatching rate of the embryo was 100%, whereas, at the highest concentration (1000 *μ*g/ml), there was no hatching observed. However, paracetamol showed a decline in hatching rate, yet delayed hatching was not seen.

### 3.4. *M. charantia* Toxic Effect on the Scoliosis of* Danio rerio* Larvae

**Figure 8:**
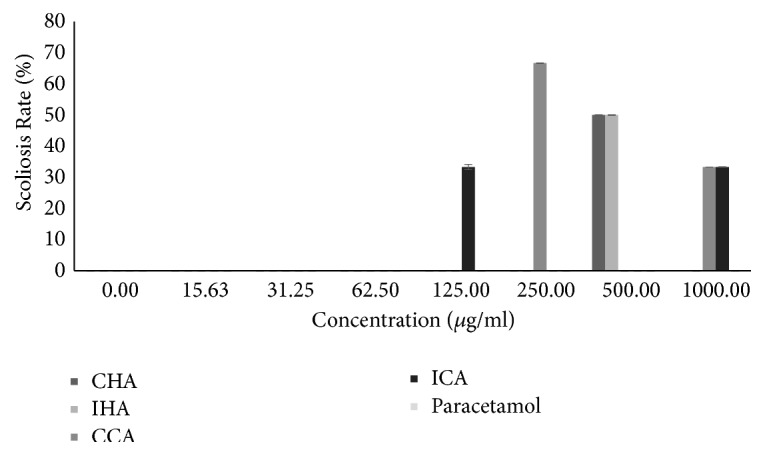
The scoliosis rate of zebrafish embryo tested on all test extracts and control solutions at different series of concentration. Results were expressed as mean ± SEM of three independent experiments performed in triplicate.

Based on the result presented (Figure 8), the scoliosis was only seen in the higher concentrations (125-1000 *μ*g/ml) of all test extracts. There was no scoliosis seen in lower concentrations (0-62.5 *μ*g/ml). The result showed that the cold aqueous Indian* M. charantia* exhibited a mild scoliosis effect on zebrafish larvae. However, the cold aqueous Chinese* M. charantia* expressed a higher scoliosis effect compared to all the other extracts. And as for both the hot the cold aqueous* M. charantia* extract, moderate scoliosis effect was observed on zebrafish larvae. No scoliosis was observed in positive control solution.

**Figure 9:**
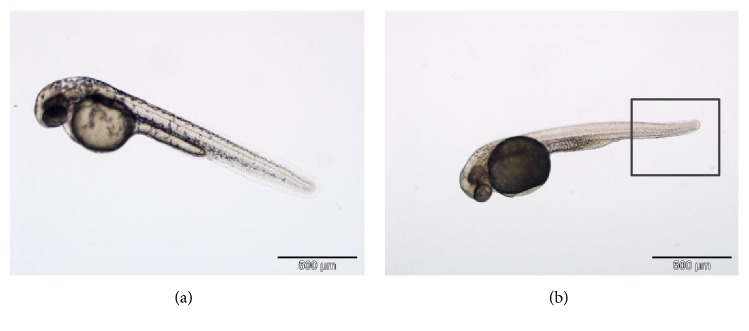
The normal appearance of hatched embryo (a) compared to the scoliosis present hatched embryo (b) at magnification of 500 *μ*m using Microscope, OLYMPUS DX51; Camera, OLYMPUS DP72.

The scoliosis present in the hatched embryo compared to the normal hatched embryo was observed as in Figure 9.

### 3.5. The Heartbeat of Zebrafish Larvae Treated with* M. charantia* Extract

**Figure 10:**
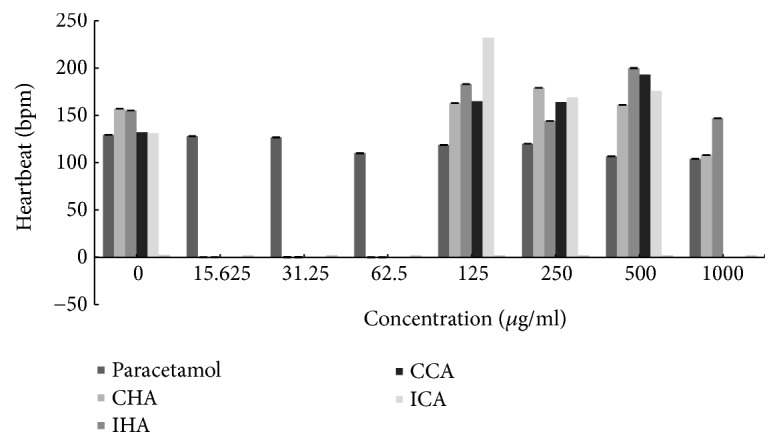
The heartbeat rate of zebrafish embryo examined on all test extracts and control solutions at various series of concentration.

All the* M. charantia* extracts presented a normal heartbeat, but at higher concentration of test extract, the heartbeat counting differed as shown in Figure 10. The hot aqueous Chinese* M. charantia *had an ideal heartbeat for all series of concentration, but at higher concentration (1000 *μ*g/ml) the heartbeat counting eventually reduced. As for the hot aqueous Indian* M. charantia*, the overall heartbeat rate of zebrafish larvae decreased inconsistently as the concentration of test extract increased, which is specifically observed at 125 *μ*g/ml until 1000 *μ*g/ml. However, only the cold aqueous Chinese* M. charantia* showed that the heart rate increased within normal range, along with the higher concentration. Lastly, cold aqueous Indian* M. charantia* showed that the heartbeat decreased but remained within the normal range, as the concentration of test extract increased. Heartbeat was stable for the positive control.

## 4. Discussion

It can be summarised that the Indian* M. charantia* reflects a higher survival rate as compared to the Chinese* M. charantia*. This is because, although both the* M. charantia* extracts have the same components, they still have different amounts of total compounds present in the pulp of the fruit of* M. charantia*. Some of the main bioactive components are polysaccharides, peptides, protein, lipids, terpenoids, saponins, phenolics, and sterols, each with specific functional properties [25]. As stated, the protein content and total phenolic content of Indian* M. charantia* flesh are higher than those of the Chinese* M. charantia* flesh [26]. This may be one of the factors which contribute to the higher survival rate of Indian* M. charantia* which makes it a healthier choice as compared to the Chinese* M. charantia*.

As there is apparent difference between the two types of aqueous extraction method, the hot aqueous extraction method expressed a lower overall survival rate than the cold aqueous extraction samples. This is because the extraction method can influence the composition of extract due to different types of reaction during the aqueous extraction procedure. Specifically, flavonoid is one of the important elements in* M. charantia* as it possesses antioxidant property which removes the excess free radicals by acting as a neutralizing agent [25]. However, flavonoids extraction is known to be more efficient when the temperature is low because the higher temperature degrades the flavonoids component despite a rapid and high rate of reaction [27]. As reported, the absence of flavonoid compound in the phytochemical screening of* M. charantia* extract was proven using hot aqueous extraction method [28]. Thus, the absence of flavonoids can cause oxidative stress indirectly damaging the cellular molecules resulting in a lower survival rate of zebrafish embryo [29].

The study of the toxicity of* M. charantia* leaf using African catfish model reported that the survival rate of catfish decreased as the level of powdered leaf increased. The effect was also seen on blood components whereby the erythrocytes and Hb count decreased, whereas leukocytes count increased, as the powdered leaf of* M. charantia* increased [30]. Thus, this showed that the* M. charantia* generally provides a low survival rate as the amount of extracts increases. Meanwhile, the paracetamol used as the positive control had a higher survival rate compared to the other test extracts. The negative control showed 100% survival rate and this indicates that the negative control was an ideal condition for the survival of zebrafish embryo. This revealed that the negative control did not show any sort of toxicity effect, whereas the positive control exhibited a lower toxic effect as compared to all the test extracts seen on the survival rate of* Danio rerio* embryo.

The Indian* M. charantia* reflects a higher LC_50_ value which indicates that it is a better choice than the Chinese* M. charantia* as it ends in a lower toxicity level. In general, the Chinese* M. charantia* has lower protein and phenolic content as compared to Indian* M. charantia* [26]. This factor can eventually influence the mortality rate of the zebrafish embryo, causing the Chinese* M. charantia* to produce more toxic effects. Additionally, the cold aqueous showed a higher LC_50_ value than the hot aqueous method for both types of species. This suggested that the cold extraction method is a superior extraction method for* M. charantia* sample preparation. Apparently, the hot aqueous extract can degrade certain enzymes and molecules such as flavonoids which can induce toxicity effect despite higher extract yields [27]. Similarly, the hot aqueous extraction produces higher extract yields compared to cold aqueous extraction. Hence, the toxic compounds like cucurbitanes will be high in hot aqueous extract, causing it to exhibit more toxic effect affecting the mortality rate of zebrafish embryo [31].

In addition, coinciding with another study, the median lethal dose of* M. charantia* ethanolic extract is safe to be consumed below 2000 mg/kg of Sprague Dawley rats [32]. It is also stated that there might be potential toxic effect on blood, tissue, and organ specifically the liver if the* M. charantia* is consumed at a higher dose [32]. The LC_50_ value of paracetamol is higher than the test extract, because the mortality rate in treatment extract is higher than the mortality rate in control solution. This may be due to the components of* M. charantia* extract such as cucurbitane that is a type of protein causing a toxic effect and may disrupt the biological pathway of zebrafish embryo halting its development [25].

Moreover, the overall hatchability of zebrafish embryo declines as the concentration of all test extracts increases. Basically, the* M. charantia* is known to cause abortion, so this point can be correlated with the delayed hatching rate of zebrafish embryo observed.* M. charantia* contains *α*-momorcharin (*α*-MMC) and *β*-momorcharin (*β*-MMC) that contribute to antifertility activity [25]. The *α*-MMC mainly causes the termination of pregnancy during early stage which indirectly leads to miscarriage, while the *β*-MMC affects the adhesion and implantation of the embryo at the same time depressing the development of the embryo [25]. Hence, this may underlie the fact that the* M. charantia* generally disrupts the hatching rate of the embryo. Likewise a previous study on* M. charantia* leaf extract showed similar results on hatching rate [33]. The same observation was shown whereby the percentage of hatching rate reduced as the* M. charantia* extract concentration increased, which implied that the normal process of zebrafish embryo hatching rate was disturbed [33]. Another type of animal model embryo study using* Fasciola hepatica*, which is a type of trematode, reported that the embryogenesis of* F. hepatica* eggs was inhibited upon exposure to* M. charantia* extract [34]. This finding provided a promising source of anthelmintic agent and antifertility properties of* M. charantia*. Next, similar to test extract, the positive control decreased the hatching rate of zebrafish along with the increase in concentration. Oppositely, the negative control showed the highest hatching rate because it is the ideal condition for normal hatching process of zebrafish embryo [25].

Scoliosis of zebrafish larvae was only observed in the higher concentration of* M. charantia* extract. In general, the scoliosis rate of* M. charantia* might be due to the high amount of toxic compound present at a higher concentration as mentioned above. This composition of* M. charantia* may alter the development of vertebrae formation which leads to failure in spine development [35]. Other than toxic compound, the teratogenic effect of* Danio rerio* larvae can be affected due to the delayed hatching rate of embryo upon exposure to* M. charantia* [36]. A similar effect of scoliosis was observed in the previous study of* M. charantia* leaf extract tested on zebrafish embryo model where the teratogenic effect of* M. charantia* leaf extract was observed at higher concentration, specifically at 0.5% concentration [33]. Nevertheless, the compound of* M. charantia* which is responsible for this effect was not identified [33]. There was no scoliosis observed in negative control as well as all series of positive control. This manifested that the negative control and positive control do not reveal any toxic effect, in particular, scoliosis effect, as compared to all the test extracts. This finding contradicts that of the previous study where the acetaminophen on* Danio rerio* embryo instigated anomalies at a higher concentration causing impairment [37].

Furthermore, a study has proven that the* M. charantia* extract is a beneficial antihypertensive agent as it indirectly reduces blood pressure by decreasing heartbeat of zebrafish larvae. The* M. charantia* lowers the heart rate by inhibiting the angiotensin-converting enzyme (ACE) which is a type of enzymes that is responsible for converting angiotensin I to angiotensin II which constricts blood vessels. The inhibition of ACE leads to blood vessel dilation reducing the blood pressure as well as lowering the workload of heart. This influences the heart rate causing the heartbeat to slow down [38]. Another study reported that whole plant extract of* M. charantia* possessed antihypertensive properties, by normalizing the heart rate of hypertensive induced rats and decreasing the systemic arterial blood pressure. It was also reported that* M. charantia* acts as an antihypertensive agent, nevertheless this was not completely investigated [39]. Another study showed that bradycardia was observed in Zucker Obese (ZO) rats upon consumption of* M. charantia *[40]. Conversely, some studies reported slight increase of heart rate in the group of lean rats upon the consumption of this extract [40]. The previous result of increased heart rate was similar to that found in the cold aqueous Chinese* M. charantia* indicating that the low heart rate can also result in tachycardia. Lastly, both types of control solutions gave stable heartbeat within the normal range compared to the* M. charantia* extract.

## 5. Conclusion

In summary, the impact of* M. charantia* exposure on zebrafish embryo model through acute toxicity assay assessment was evaluated. A comparison of two different extraction methods showed that the cold aqueous extraction method manifested lower toxic effects compared to hot aqueous extraction. The Indian* M. charantia* was observed to possess a milder toxic effect compared to the Chinese* M. charantia*. Therefore, the results conclude that the cold aqueous Indian* M. charantia* is the best test extract as it exhibited lower toxicity effect in overall parameters such as survival rate, LC_50_ value determination, hatching rate, scoliosis, and heartbeat of the zebrafish embryo. Hence, although this plant extract is safe to be consumed due to its promising pharmaceutics, it still exhibits mild toxicity effect on a higher concentration as evaluated in the zebrafish embryo model. Thus, it is suggested that further experimental study on phytochemical screening should be conducted to identify the specific components of* M. charantia* which exhibit respective toxic effects.

## Data Availability

The data used to support the findings of this study are available from the corresponding author upon request.
